# Supplementary material for: RADpainter and fineRADstructure: Population Inference from RADseq Data
Source: Mol Biol Evol. 2018 Feb 20;35(5):1284–90. doi: 10.1093/molbev/msy023 (PMC5913677; doi:10.1093/molbev/msy023)
Supplement: Supplementary Data [file msy023_supp.pdf]

Algorithm 1: Coancestry estimation: a simple case with haploid individuals and without missing data

Data: For each locus, the sequences of nucleotides that vary between individuals

Result: Estimated coancestry matrix

Init: Initialize the coancestry matrix $\mathbf{C}$, so that $\mathbf{C}_{ij}$=0 for all i and j

```
foreach RAD locus do
    foreach individual i // a recipient
    do
        foreach individual j ≠ i // potential donors
        do
            D_ij = number of SNPs between i and j;
        end
        M_i = min_{j≠i}(D_ij); // The minimum of D_ij for all j≠i
        N_Mi = count_{j≠i}(D_ij==M_i); // Count the number of ‘donors’
        foreach individual j ≠ i do
            if (D_ij == M_i) // If individuals i and j are closest, j is a ‘donor’
            then
                // Assign an equal proportion of coancestry to each donor
                C_ij = C_ij + 1/N_Mi
            end
        end
    end
end
```
